# *MuMADS1* and *MaOFP1* regulate fruit quality in a tomato *ovate* mutant

**DOI:** 10.1111/pbi.12843

**Published:** 2017-11-02

**Authors:** Juhua Liu, Jing Zhang, Jingyi Wang, Jianbin Zhang, Hongxia Miao, Caihong Jia, Zhuo Wang, Biyu Xu, Zhiqiang Jin

**Affiliations:** ^1^ Key Laboratory of Tropical Crop Biotechnology Ministry of Agriculture Institute of Tropical Bioscience and Biotechnology Chinese Academy of Tropical Agricultural Sciences Haikou China; ^2^ Key Laboratory of Genetic Improvement of Bananas Hainan Province Haikou Experimental Station Chinese Academy of Tropical Agricultural Sciences Haikou China

**Keywords:** *MuMADS1*, *MaOFP1*, fruit quality, regulation, tomato *ovate* mutant

## Abstract

Fruit ripening and quality are common botanical phenomena that are closely linked and strictly regulated by transcription factors. It was previously discovered that a banana MADS‐box protein named MuMADS1 interacted with an ovate family protein named MaOFP1 to regulate banana fruit ripening. To further investigate the role of *MuMADS1* and *MaOFP1* in the regulation of fruit quality, a combination of genetic transformation and transcriptional characterization was used. The results indicated that the co‐expression of *MuMADS1* and *MaOFP1* in the *ovate* mutant could compensate for fruit shape and inferior qualities relating to fruit firmness, soluble solids and sugar content. The number of differentially expressed genes (DEGs) was 1395 in WT vs. *ovate*, with 883 up‐regulated and 512 down‐regulated genes, while the numbers of DEGs gradually decreased with the transformation of *MuMADS1* and *MaOFP1* into *ovate. ‘*Starch and sucrose metabolism’ constituted the primary metabolic pathway, and the gene numbers in this pathway were obviously different when *MuMADS1* and *MaOFP1* were integrated into *ovate*. A series of metabolic genes involved in cell wall biosynthesis were up‐regulated in the WT vs. *ovate*, which probably resulted in the firmer texture and lower sugar contents in the *ovate* fruit. These results demonstrate that *MuMADS1* and *MaOFP1* are coregulators of fruit quality, facilitating the dissection of the molecular mechanisms underlying fruit quality formation.

## Introduction

MADS‐box genes play important roles in nearly every aspect of plant growth and development (Benlloch *et al*., 2009; Ferrario *et al*., 2006; Kaufmann *et al*., 2009; Liu *et al*., 2013). MADS‐box transcription factors (TFs) are considered to be the most powerful TFs identified for regulating fruit development and ripening (Karlova *et al*., 2014). Most investigations regarding the function of MADS‐box TFs in fruit development and ripening have focused on the model plant tomato (*Solanum lycopersicum*), while little information is available about other plants, particularly the important tropical fruit banana.

MADS‐box transcription factors act via a complex network of protein–DNA and homo‐ or heterodimeric protein–protein interactions (Tonaco *et al*., 2006). MADS‐box TFs bind other proteins through the K‐domain (Kaufmann *et al*., 2005; Liu *et al*., 2015a,b; Smaczniak *et al*., 2012a). These TFs often bind as multimers. Subsequently, the two dimers interact to form a tetramer and loop the DNA that lies between the two CArG boxes (Melzer *et al*., 2009; Smaczniak *et al*., 2012b). These complexes contain not only MADS domain proteins, but also other non‐MADS‐box proteins such as SEUSS, histone fold protein NF‐YB and ubiquitin‐activating (UBA) enzyme E1 protein MuUBA (Liu *et al*., 2013; Masiero *et al*., 2002; Sridhar *et al*., 2006).

The *OVATE* gene was first cloned by positional cloning in tomato and was demonstrated to encode a hydrophilic protein with a putative bipartite nuclear localization signal and a C‐terminal domain of approximately 70 amino acids, which was designated as the OVATE domain, indicating that the gene belongs to the ovate family of proteins (OFP) (Liu *et al*., 2002; Wang *et al*., 2011; Yu *et al*., 2015). As a member of a plant‐specific transcription factor family, the OVATE protein was first identified in tomato as the prime controller of fruit appearance (Ku *et al*., 1999; Liu *et al*., 2002; Monforte *et al*., 2014; Tsaballa *et al*., 2011; Wang *et al*., 2015; Wu *et al*., 2015). The impacts of the *ovate* mutation on fruit appearance differ from elongation to creating pear‐ or globe‐shaped fruits according to the genetic context with the *ovate* modification (Gonzalo and van der Knaap, 2008). This indicates that OVATE cannot be held accountable for the noted phenotype and likely is in contact with various genes in an epistatic way (Azzi *et al*., 2015). This ovate pear‐formed phenotype was enhanced by a genomic DNA fragment containing the *OVATE* gene as well as its ectopic overexpression, causing its reversion to the production of globe‐shaped fruits (Liu *et al*., 2002). Thus, the *ovate* modification is probably a loss‐of‐function mutation from a negative controller of plant development whose purpose needs to be determined (Azzi *et al*., 2015). A transcriptional activity examination of *Arabidopsis* OFPs (AtOFPs) in protoplasts indicates that they function as transcription repressors (Wang *et al*., 2007, 2011). Functional categorization of OFPs from various plant species, such as *Arabidopsis*, rice, tomato, melon and pepper, implies that OFPs control numerous portions of plant initiation and maturation, including ovule progression (Pagnussat *et al*., 2007), vascular progression (Schmitz *et al*., 2015) and secondary cell wall development (Li *et al*., 2011), which is probably made via the connection with various kinds of TFs, such as KNOX and BELL classes, and/or via immediate control of the expression of target genes, namely *gibberellin‐20‐oxidase* (*GA20ox*) and *GSK3‐like kinase* (Hackbusch *et al*., 2005; Wang *et al*., 2016; Yang *et al*., 2016).

We previously confirmed a novel role for an OFP in regulating banana fruit ripening. The banana OFP1 (MaOFP1) interacted with a banana MADS‐box protein MuMADS1 to take on antagonistic parts in ethylene‐prompted postharvest maturation in banana (Liu *et al*., 2015b). However, the banana fruit ripening process also constitutes a process for banana fruit quality formation. Whether *MuMADS1* and *MaOFP1* possess novel roles in fruit quality formation remains unclear. Herein, *MuMADS1* and *MaOFP1* were transformed into a tomato *ovate* mutant, and the phenotype and physiology of the transformants were analysed. The function of *MuMADS1* and *MaOFP1* in regulating fruit quality was further investigated by transcriptional analysis.

## Results

### Identification of transgenic plants

To additionally examine the parts played by *MuMADS1* and *MaOFP1*, 35S: *MuMADS1* (pCAMBIA1302‐*MuMADS1*), 35S: *MaOFP1* (pCAMBIA1302‐ *MaOFP1*) and 35S: *MuMADS1*+35S: *MaOFP1* were transformed into tomato *ovate* mutants. More than three independent transgenic lines confirmed for transgene integration were recovered, and two independent lines for each transformant were chosen for their transgene homozygosity in the T1 generation from the DNA gel blot evaluation and were phenotypically categorized in T3 production (Figure S1a–c). *MuMADS1* expression in different fruit development stages [immature green(IMG), mature green (MG), breaker fruit (BR) and red mature (RM)] of the four transgenic lines (M1 and M2 for 35S: *MuMADS1* transformants, MO1 and MO2 for 35S: *MuMADS1 *+ 35S: *MaOFP1* transformants) and *MaOFP1* expression in various fruit maturation periods of the four transgenic lines (O1 and O2 for 35S: *MaOFP1* transformants, MO1 and MO2 for 35S: *MuMADS1 *+ 35S: *MaOFP1* transformants) was additionally determined via quantitative real‐time PCR (qRT‐PCR). The outcomes suggested that *MuMADS1* and *MaOFP1* were greatly expressed in transgenic tomatoes. During the process of fruit development, the gene expression levels gradually increased and peaked at the RM stage. Moreover, *MuMADS1* and *MaOFP1* were expressed at higher levels in MO transgenic lines than in the M or O transgenic lines (Figure S1d and e). These results suggested that *MuMADS1* and *MaOFP1* had been incorporated into the tomato *ovate* genome and exhibited steady expression.

### Regulation of *MuMADS1* and *MaOFP1* on fruit shape and quality

**Figure 1:**
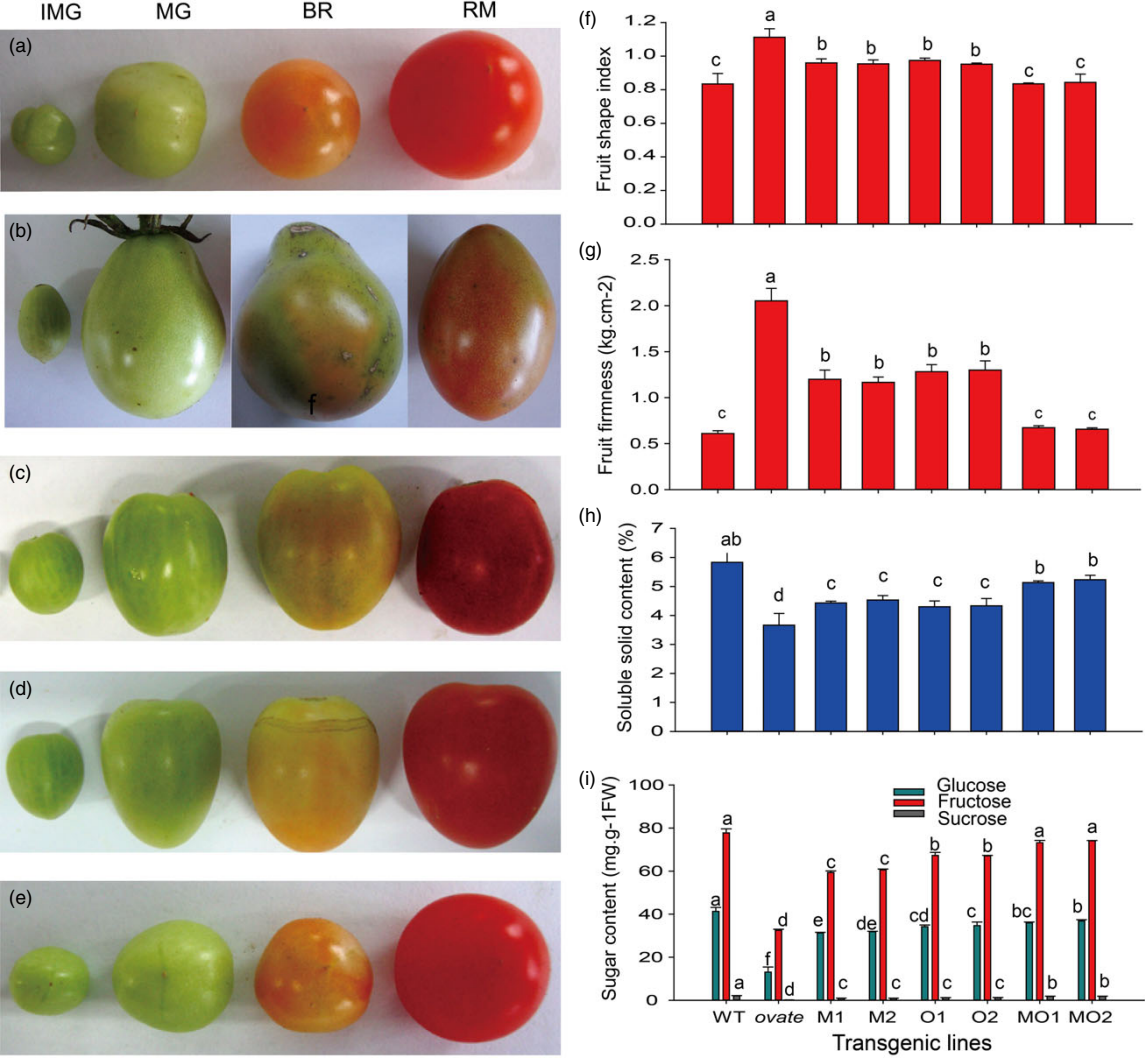
Effect of *MuMADS1* and *MaOFP1* on fruit shape and quality. (a–e), phenotype of WT and transgenic lines at IMG, MG, BR and RM stages. (a) WT; (b) *ovate* mutation; (c) *MuMADS1‐*transformed fruits; (d) *MaOFP1‐*transformed fruits; (e) *MuMADS1 *+* MaOFP1‐*transformed fruits; (f) fruit firmness; (g) soluble solids; (h) sugar contents; (i) organic acids. The fruits for f, g, h and i are at the RM stage. M1 and M2, two *MuMADS1‐*transformed lines; O1 and O2, two *MaOFP1‐*transformed lines; MO1 and MO2, *MuMADS1 *+* MaOFP1‐*transformed lines.

The obvious phenotype of the transgenic line is a change in fruit shape. The fruit shape of WT is round, and the *ovate* mutant is pear‐shaped, while *MuMADS1‐*transformed or *MaOFP1‐*transformed plants had oblong fruit, which represents a partial recovery of the fruit shape. However, when *MuMADS1* and *MaOFP1* were cotransformed into the *ovate* mutant, the fruit shape changed to round, which represented a full recovery of the fruit shape. This result demonstrated that fruit shape was regulated to a large extent by *MuMADS1* and *MaOFP1* (Figure 1a–f).

The effects of *MuMADS1* and *MaOFP1* on fruit quality including fruit firmness, soluble solids, sugar and organic acids were also further analysed. As shown in Figure 1g, the fruit firmness of WT at the red mature stage was 0.61 kg/cm^2^, while in *ovate,* it dramatically increased to 2.05 kg/cm^2^. For *MuMADS1‐* or *MaOFP1‐*transformed plants, the firmness decreased to 1.2 and 1.3 kg/cm^2^, respectively. For the *MuMADS1* and *MaOFP1* cotransformed plants, the firmness decreased to 0.65 kg/cm^2^, representing recovery of the firmness quality. The soluble solid content of WT in the red mature fruit was 4.9%, while in the *ovate* mutant, it was reduced to 3.7%. For *MuMADS1‐* or *MaOFP1‐*transformed plants, the soluble solid content increased to 4.2% and 4.3%, respectively. For *MuMADS1* and *MaOFP1* cotransformed plants, the soluble solids content increased to more than 5.0%, indicating full recovery (Figure 1h). In WT tomato fruit, the contents of fructose, glucose and sucrose were 77.74, 41.27 and 2.10 mg/g FW, respectively, while the *ovate* mutation reduced these to 32.48, 13.10 and 0.16 mg/g FW, respectively. For *MuMADS1‐*transformed plants, the contents of fructose, glucose and sucrose were increased to approximately 59.34, 31.27 and 0.94 mg/g FW, respectively. For *MaOFP1‐*transformed plants, the contents of fructose, glucose and sucrose increased to more than 67.28, 34.18 and 1.21 mg/g FW, respectively, which represents a partially recovered sugar content. For *MuMADS1* and *MaOFP1* cotransformed plants, the contents of fructose, glucose and sucrose were increased to approximately 73.21, 35.97 and 1.85 mg/g FW, respectively, which suggests that the sugar content was mostly recovered (Figure 1i). These results suggested that *MuMADS1* and *MaOFP1* compensate for the phenotype of the *ovate* mutation and regulate fruit shape and quality. Furthermore, the co‐expression of *MuMADS1* and *MaOFP1* in the *ovate* mutation could enhance the quality of the fruit in comparison with the separate transformation of *MuMADS1* or *MaOFP1*.

### RNA‐Seq analysis

A total of 416.06 million clean reads from the samples were obtained following quality assessment and data filtering. The GC% of sequenced data from the 15 libraries was 42.56%, and the percentage of reads with an average quality score >30 was 88.36%. This indicated that the accuracy and quality of the sequencing data were sufficient for further analysis. The general sequencing statistics are shown in Table S1. The mapping efficiency of 15 samples to the tomato (*S. lycopersicum)* genome was ~80.92%–85.62%, as shown in Table S2.

### Number of differentially expressed genes (DEGs) in transgenic fruits

**Figure 2:**
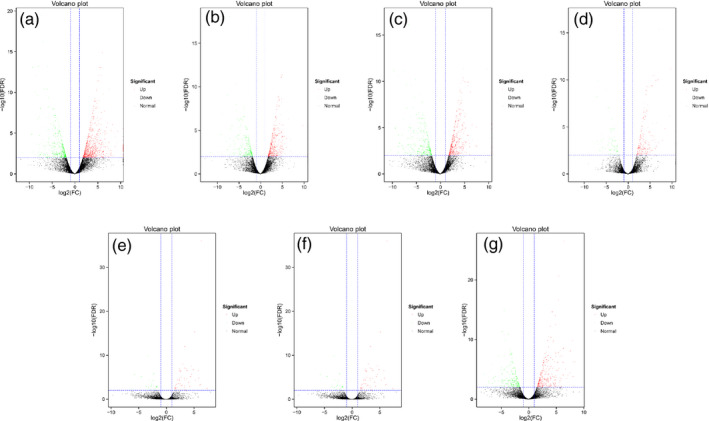
Number of DEGs in transgenic fruits. (a) WT vs. *ovate*; (b) WT vs. *MuMADS1* transformants; (c) WT vs. *MaOFP1* transformants; (d) WT vs. *MuMADS1 + MaOFP1* transformants; (e) *ovate* vs. *MuMADS1* transformants; (f) *ovate* vs. *MaOFP1* transformants; (g) *ovate* vs. *MuMADS1 + MaOFP1* transformants.

To further investigate the mechanism of *MuMADS1* and *MaOFP1* in regulating fruit quality, transcriptional analysis was considered. The number of DEGs was 1395 in WT vs. *ovate,* with 883 up‐regulated and 512 down‐regulated genes (Figure 2a), while the DEGs in the WT vs. *MuMADS1*‐transformed fruits and *MaOFP1‐*transformed fruits included 842 (502 up and 340 down) and 904 (500 up and 404 down), respectively (Figure 2b and c). The number of DEGs was only 286 in WT vs. *MuMADS1 *+* MaOFP1*‐transformed fruits, with 209 up‐regulated and 77 down‐regulated genes (Figure 2d). Conversely, the DEGs in the *ovate* vs. *MuMADS1*‐transformed fruits and *MaOFP1‐*transformed fruits were 84 (58 up and 26 down) and 342 (135 up and 207 down), respectively (Figure 2e and f), and 530 (287 up and 243 down) in the *ovate* vs. *MuMADS1 *+* MaOFP1*‐transformed fruits (Figure 2g). These results suggest that the largest number of DEGs existed in WT vs. *ovate*. With the transformation of *MuMADS1* or *MaOFP1* into *ovate*, the DEGs in the WT vs. transformed fruits decreased. When *MuMADS1* and *MaOFP1* were co‐expressed in *ovate*, the DEGs were reduced to their lowest number. Alternatively, with the transformation of *MuMADS1* or *MaOFP1* into *ovate*, the DEGs in the *ovate* vs. transformed fruits increased. When *MuMADS1* and *MaOFP1* were co‐expressed in *ovate*, the DEGs in the *ovate* vs. transformed fruits increased the most. These results suggest that the expression of *MuMADS1* and *MaOFP1* in *ovate* reduces the differences in expressed genes in WT vs. transformants and increases the differences in expressed genes in *ovate* vs. transformants. Briefly, *MuMADS1* and *MaOFP1* can regulate and recover the DEGs in *ovate*.

### Gene annotation and function classification

**Figure 3:**
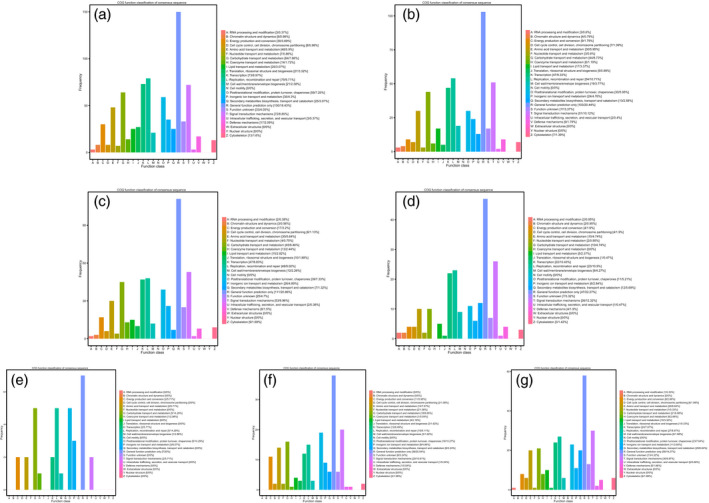
Histogram of COG classification. (a) WT vs. *ovate*; (b) WT vs. *MuMADS1* transformants; (c) WT vs. *MaOFP1* transformants; (d) WT vs. *MuMADS1 + MaOFP1* transformants; (e) *ovate* vs. *MuMADS1* transformants; (f) *ovate* vs. *MaOFP1* transformants; (g) *ovate* vs. *MuMADS1 + MaOFP1* transformants.

A total of 4253 DEGs were obtained from seven assemblies, and 437 of the genes were novel. The nonredundant (Nr), Swiss‐Prot, Gene Ontology (GO), Clusters of Orthologous Groups (COG), Eukaryotic Orthologous Groups (KOG), evolutionary genealogy of genes: Nonsupervised Orthologous Groups (eggNOG) and Kyoto Encyclopedia of Genes and Genomes (KEGG) databases were introduced to annotate the DEGs. Of these, 4252 (99.98%) were matched to the Nr database, while the smallest number of DEGs was found in the KEGG database (Table S3). To further appraise the completeness of the RNA‐Seq data, COG classifications were performed with the DEGs. In the assembly of the WT vs. *ovate* mutant, a total of 557 DEGs were annotated in 25 COG categories (Figure 3a). The category of ‘general function prediction only’ (150, 18.43%) ranked highest, followed by ‘replication, recombination and repair’ (79, 9.71%) and ‘transcription’ (73, 8.97%). The ‘signal transduction mechanism’ (72, 8.85%) and ‘carbohydrate transport and metabolism’ (64, 7.86%) categories ranked fourth and fifth, respectively. With the transformation of *MuMADS1* or *MaOFP1* into the *ovate* mutant, the DEGs in WT vs. transformed fruits decreased. For example, in the assemblies of WT vs. *MuMADS1* transformants and WT vs. *MaOFP1* transformants, the category of ‘carbohydrate transport and metabolism’ decreased to 44 (8.73%) and 45 (8.46%), respectively (Figure 3b and c), while in the assembly of WT vs. *MuMADS1 + MaOFP1* transformants, it decreased to 10 (4.74%; Figure 3d). Conversely, with the transformation of *MuMADS1* or *MaOFP1* into the *ovate* mutant, the number of DEGs in *ovate* vs. the transformed fruit gradually increased. For example, in the assemblies of *ovate* vs. *MuMADS1* transformants, *ovate* vs. *MaOFP1* transformants and *ovate* vs. *MuMADS1 + MaOFP1* transformants, the category of ‘carbohydrate transport and metabolism’ gradually increased to five (14.29%), 16 (8.65%) and 21 (6.89%), respectively (Figure 3e, f and g).

Unigenes that were successfully annotated to the GO database were classified into three principal categories for the seven assemblies, including ‘cellular component’, ‘molecular function’ and ‘biological process’, which were further subdivided into 53 categories (Table S4; Figure S2). Of these, the categories that were most represented in the ‘biological process’ principal category included ‘immune system process’ [WT vs. *ovate*: 10 (0.40%); WT vs. *MuMADS1* transformants: eight (0.32%); WT vs. *MaOFP1* transformants: eight (0.32%); WT vs. *MuMADS1 *+* MaOFP1* transformants: six (0.24%); *ovate* vs. *MuMADS1* transformants: 0; *ovate* vs. *MaOFP1* transformants: 0; *ovate* vs. *MuMADS1 *+* MaOFP1* transformants: six (0.24%)], followed by ‘signalling’ [WT vs. *ovate*: 13 (0.30%; WT vs. *MuMADS1* transformants: 10 (0.23%); WT vs. *MaOFP1* transformants: 11 (0.26%); WT vs. *MuMADS1 *+* MaOFP1* transformants: 10 (0.23%); *ovate* vs. *MuMADS1* transformants: 0; *ovate* vs. *MaOFP1* transformants: one (0.02%); *ovate* vs. *MuMADS1 *+* MaOFP1* transformants: six (0.14%)]. Within the ‘cellular component’ principal category, unigenes [WT vs. *ovate*: one (2.13%); WT vs. *MuMADS1* transformants: 0; WT vs. *MaOFP1* transformants: one (2.13%); WT vs. *MuMADS1 *+* MaOFP1* transformants: 0; *ovate* vs. *MuMADS1* transformants: 0; *ovate* vs. *MaOFP1* transformants: one (2.13%); *ovate* vs. *MuMADS1 *+* MaOFP1* transformants: 0] belonged to the ‘extracellular matrix’ category. In the principal category of ‘molecular function’, the most‐represented category was ‘enzyme regulator activity’ [WT vs. *ovate*: two (0.51%); WT vs. *MuMADS1* transformants: two (0.51%); WT vs. *MaOFP1* transformants: one (0.26%); WT vs. *MuMADS1 *+* MaOFP1* transformants: one (0.26%); *ovate* vs. *MuMADS1* transformants: 0; *ovate* vs. *MaOFP1* transformants: 0; *ovate* vs. *MuMADS1 *+* MaOFP1* transformants: 0].

### Pathway enrichment analysis of DEGs

**Figure 4:**
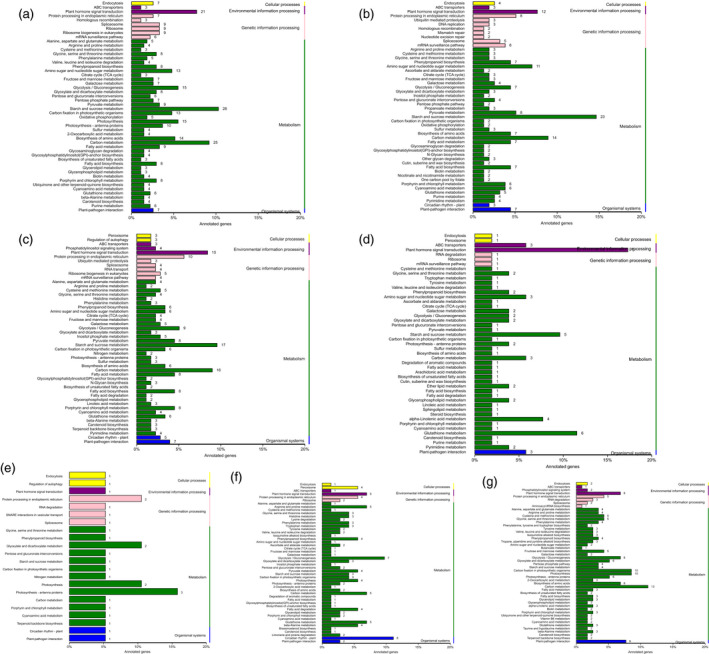
Histogram of KEGG pathway classification. (a) WT vs. *ovate*; (b) WT vs. *MuMADS1* transformants; c, WT vs. *MaOFP1* transformants; (d) WT vs. *MuMADS1 + MaOFP1* transformants; (e) *ovate* vs. *MuMADS1* transformants; (f) *ovate* vs. *MaOFP1* transformants; (g) *ovate* vs. *MuMADS1 + MaOFP1* transformants.

To identify the active pathways in the transformants, the obtained unigenes were mapped to the canonical reference pathways in the KEGG database. KEGG pathway enrichment examination was performed to classify the biological tasks of the DEGs. Specific enrichment of genes was obtained for 98, 85, 88, 47, 22, 61 and 74 pathways for WT vs. *ovate*, WT vs. *MuMADS1* transformants, WT vs. *MaOFP1* transformants, WT vs. *MuMADS1 + MaOFP1* transformants, *ovate* vs. *MuMADS1* transformants, *ovate* vs. *MaOFP1* transformants and *ovate* vs. *MuMADS1 + MaOFP1* transformants, respectively (Table S5). The top 50 enriched pathways are displayed in Figure 4. ‘Starch and sucrose metabolism’ was the most frequently noted and had 28 (11.07%) DEGs in the WT vs. *ovate*, 23 (9.09%) DEGs in WT vs. *MuMADS1* transformants, 16 (6.32%) DEGs in WT vs. *MaOFP1* transformants, five (1.98%) DEGs in WT vs. *MuMADS1 + MaOFP1* transformants, one (0.40%) DEG in *ovate* vs. *MuMADS1* transformants, three (1.19%) DEGs in *ovate* vs. *MaOFP1* transformants and four (1.58%) DEGs in *ovate* vs. *MuMADS1 + MaOFP1* transformants, respectively. In the assembly of WT vs. *ovate*, the second largest term was ‘carbon metabolism’, which included 25 (8.68%) DEGs. In the assemblies of WT v *MuMADS1* transformants and WT vs. *MuMADS1 + MaOFP1* transformants, the ‘carbon metabolism’ term changed from 14 (4.86) and three (1.04%) DEGs, respectively. This result indicated that ‘starch and sucrose metabolism’ and ‘carbon metabolism’ constituted the primary metabolic pathways that they changed with the integration of exogenous genes and that they might be more active during tomato fruit quality formation.

### Gene expression

**Figure 5:**
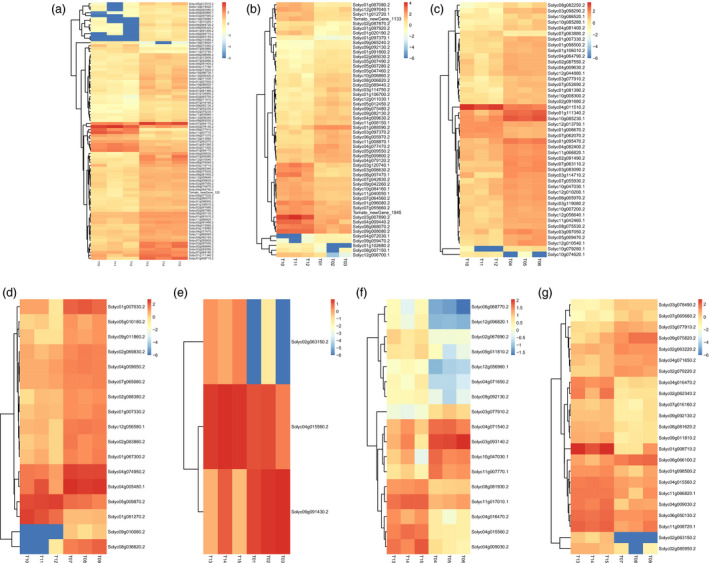
Heatmap of DEGs. (a) WT vs. *ovate*; (b) WT vs. *MuMADS1* transformants; (c) WT vs. *MaOFP1* transformants; (d) WT vs. *MuMADS1* + *MaOFP1* transformants; (e) ovate vs. *MuMADS1* transformants; (f) ovate vs. *MaOFP1* transformants; (g) ovate vs. *MuMADS1* + *MaOFP1* transformants. T10, T11, T12, three individual plants of WT; T13, T14, T15, three individual plants of *ovate* mutant; T01, T02, T03, three lines of *MuMADS1* transformants; T04, T05, T06, three lines of *MaOFP1* transformants; T07, T08, T09, three lines of *MuMADS1* + *MaOFP1* transformants.

Setting a *P *<* *0.01 and |log2 (fold change)| ≥1, the DEGs of the assemblies involved in sugar and cell wall metabolism were identified as being up‐regulated or down‐regulated (Table S6). In the WT vs. *ovate* assembly, 73 genes were up‐regulated and 13 genes were down‐regulated. When the *ovate* mutant was transformed, the DEGs gradually decreased. There were 28, 39 and 15 genes that were up‐regulated, and 26, 6, and 2 genes were down‐regulated in the assemblies of WT vs. *MuMADS1* transformants, WT vs. *MaOFP1* transformants and WT vs. *MuMADS1 *+* MaOFP1* transformants, respectively. Furthermore, the DEGs gradually increased in the assemblies of *ovate* vs. *MuMADS1‐*transformants, *ovate* vs. *MaOFP1* transformants and *ovate* vs. *MuMADS1 *+* MaOFP1* transformants with one, five and eight up‐regulated, and two, 12 and 15 down‐regulated genes, respectively (Table S6; Figure 5).

Specifically, of the 86 DEGs, 45 involved in sugar metabolism, including the *lysosomal beta‐glucosidase‐like* (*Solyc04g015560.2*), *sucrose synthase 2‐like* (*Solyc09g098590.2*), *bidirectional sugar transporter SWEET14‐like* (*Solyc03g097560.2*), were differentially expressed in the WT vs. *ovate* assembly, which suggested that these genes might be the targets of OVATE in the regulation of fruit sugar accumulation. The 41 DEGs involved in cell wall metabolism, including *cellulose synthase A catalytic subunit 5* (*Solyc11g005560.1*) and *galacturonosyltransferase 8‐like* (*Solyc12g013560.1*), were differentially expressed in the WT vs. *ovate* assembly, which suggested that these genes might be the putative targets of OVATE in the regulation of fruit firmness (Tables S6, S7).

**Figure 6:**
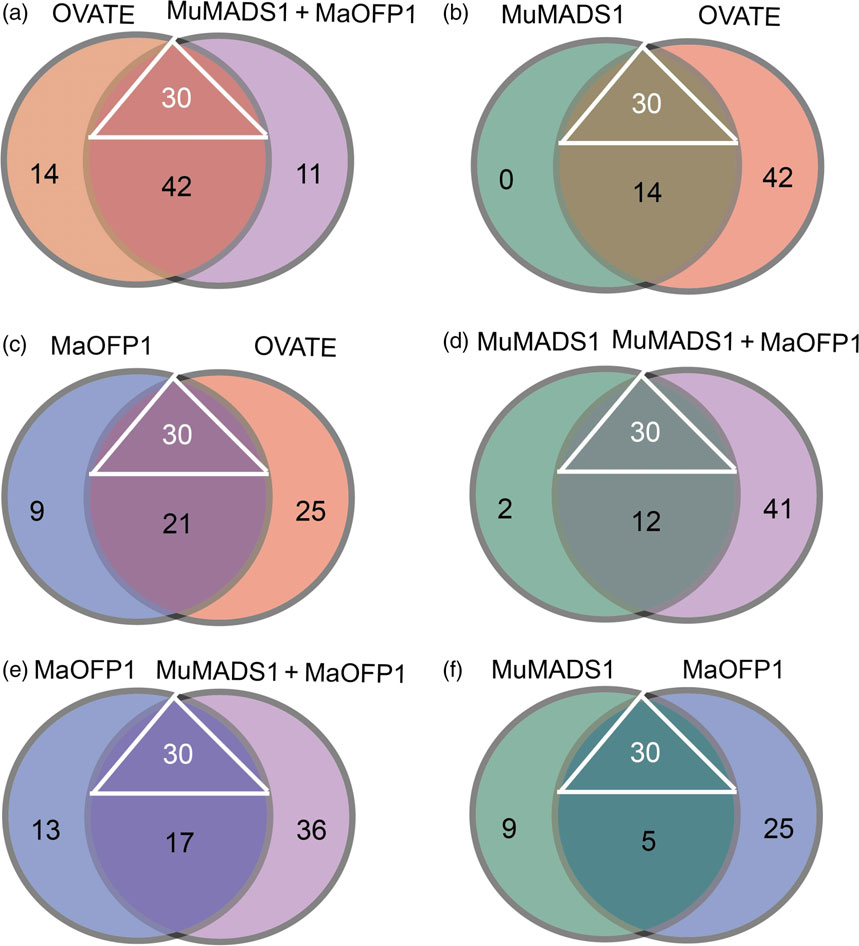
Distribution of DEGs regulated by MuMADS1, MaOFP1, MuMADS1 + MaOFP1 and OVATE. (a–f), Venn diagrams showing the overlap between the OVATE and MuMADS1 + MaOFP1, MuMADS1 and OVATE, MaOFP1 and OVATE, MuMADS1 and MuMADS1 + MaOFP1, MaOFP1 and MuMADS1 + MaOFP1, MuMADS1 and MaOFP1, respectively. The triangle indicates the 30 genes coregulated by MuMADS1, MaOFP1, MuMADS1 + MaOFP1 and OVATE.

Considering the compensation of *MuMADS1*,* MaOFP1* and *MuMADS1 *+* MaOFP1* in the *ovate* mutant, the putative target genes regulated by MuMADS1, MaOFP1 and MuMADS1 + MaOFP1 should constitute DEGs that do not overlap between the WT vs. *ovate* and the WT vs. *MuMADS1* transformants, WT vs. *ovate* and the WT vs. *MaOFP1* transformants, and the WT vs. *ovate* and the WT vs. *MuMADS1 *+* MaOFP1* transformants. Therefore, the DEGs regulated by *MuMADS1*,* MaOFP1* and *MuMADS1 *+* MaOFP1* included 44 DEGs (25 DEGs involved in carbohydrate metabolism and 19 involved in cell wall metabolism), 60 DEGS (29 DEGs involved in carbohydrate metabolism and 31 involved in cell wall metabolism) and 83 DEGs (44 DEGs involved in carbohydrate metabolism and 39 involved in cell wall metabolism), respectively. As shown in Figure 6 and Table S7, the coregulated DEGs regulated by OVATE, MuMADS1, MaOFP1 and MuMADS1 + MaOFP1 included 30 DEGs, with 14 DEGs involved in sugar metabolism and 16 involved in cell wall metabolism. The specific DEGs regulated by OVATE, MuMADS1, MaOFP1 and MuMADS1 + MaOFP1 were 56, 14, 30 and 53, respectively. Moreover, the numbers of coregulated DEGs between the OVATE and MuMADS1 + MaOFP1, MuMADS1 and OVATE, MaOFP1 and OVATE, MuMADS1 and MuMADS1 + MaOFP1, MaOFP1 and MuMADS1 + MaOFP1, MuMADS1 and MaOFP1 were 72, 44, 51, 42, 47 and 35, respectively (Figure 6; Table S7).

### Verification of gene expression by qRT‐PCR

**Figure 7:**
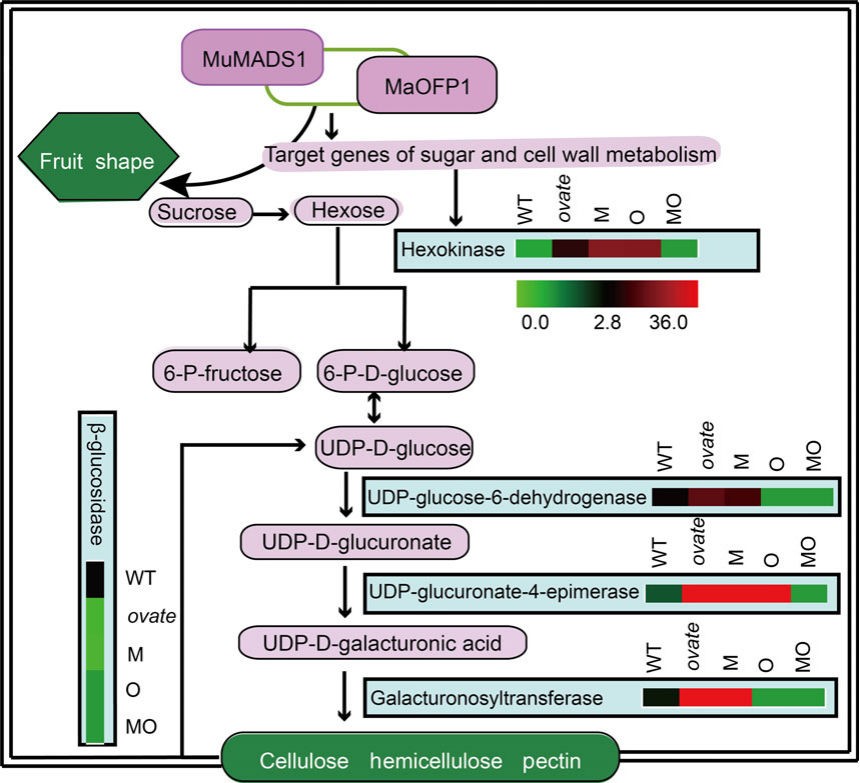
Selected members of DEGs involved in sugar and cell wall metabolism and their differential expression in transgenic lines by qRT‐PCR. The colour scale (representing log fold change values) is shown at each step.

To confirm the RNA‐Seq results, five DEGs involved in sucrose and cell wall metabolism were selected for qRT‐PCR analysis, including *plastidic hexokinase* (*Solyc04g081400.2*), putative *beta‐glucosidase 41* (*Solyc07g063880.2*), *UDP–glucose 6‐dehydrogenase 1* (*Solyc02g067080.2*), *UDP–glucuronate 4‐epimerase 1‐like* (*Solyc12g010540.1*) and *galacturonosyltransferase* (*Solyc06g083310.2*). The expression levels of the selected DEGs revealed by qRT‐PCR were generally consistent with those from the RNA‐Seq analysis at the three developmental stages, which indicated that the results of the RNA‐Seq analysis showed a high degree of correlation with those of the qRT‐PCR (Figure 7).

## Discussion

Fruit shape and quality are common botanical phenomena in nature. Banana and tomato are typical climacteric fruits. To some extent, they enjoy a common quality formation mechanism whereby the peculiar fruit quality properties can be strongly modified by TFs. The quality of tomato fruit is defined by a set of properties, including fruit appearance, flavour and texture. Flavour is defined as the combination of taste and odour. An intense taste is the result of an increase in gluconeogenesis, hydrolysis of polysaccharides, a decrease in acidity and accumulation of sugars (Prasanna *et al*., 2007), while textural characteristics are primary controlled by the cell wall structure in addition to cuticle properties, cellular turgor and fruit morphology (Vicente *et al*., 2007). In recent years, the quality of tomato fruit has been investigated at both the genetic and biochemical levels to obtain new varieties with improved taste (Carli *et al*., 2011; Causse *et al*., 2003; D'Esposito *et al*., 2017).

*OVATE* was the first fruit shape gene identified by positional cloning (Ku *et al*., 1999; Liu *et al*., 2002). Changes in the fruit shape of the *ovate* tomato mutant have been frequently studied (Monforte *et al*., 2014; Tsaballa *et al*., 2011; Wang *et al*., 2015; Wu *et al*., 2015), but little to no research has focused on fruit quality. To date, this constitutes the first report on the role of *OVATE* in regulating fruit firmness and sugar accumulation. We transformed *MuMADS1* and *MaOFP1* into tomato *ovate* plants, and the formation of transgenic lines was confirmed by Southern blotting and mRNA transcription‐level measurements (Figure S1). The fruit phenotype and quality detection results indicated that the co‐expression of *MuMADS1* and *MaOFP1* could regulate fruit shape and quality, which was consistent with the reports that the OVATE protein and MADS‐box protein regulate fruit shape and quality (Ireland *et al*., 2013; Karlova *et al*., 2014; Wang *et al*., 2015). However, the DEGs involved in regulating fruit shape were not enriched in the present study. This may be attributed to the fact that fruit shape patterning by MuMADS1 or MaOFP1 is established well before anthesis, and the DEGs regulated by MuMADS1 or MaOFP1 could not be detected at the RM stage. This is consistent with the reports of van der Knaap and Tanksley (2001), which showed that the ovary is very elongated at the time of anthesis and gradually becomes less elongated during fruit development. Additionally, Liu *et al*. (2002) also discovered that the *OVATE* transcript can be detected in tomato flowers 10 days before anthesis and begins to decrease in developing fruit 8 days after anthesis.

The fruit quality of the *ovate* mutant was poor, with the fruit firmness and soluble solids decreased (Figure 1), which was compensated for by the transformation of *MuMADS1* and *MaOFP1* into the *ovate* mutant. This result was further demonstrated by the KEGG enrichment analysis, whereby ‘starch and sucrose metabolism’ and ‘carbon metabolism’ were the primary metabolic pathways. The changed number of DEGs in the ‘starch and sucrose metabolism’ and ‘carbon metabolism’ pathways may have resulted in the inferior quality of the *ovate* tomato fruit.

The results of the de novo transcriptome analysis demonstrated that *MuMADS1* and *MaOFP1* could compensate for the phenotype of *ovate* in two ways. Firstly, the total number of DEGs in the WT vs. *ovate* mutation ranked highest (Figure 1), which suggests that the *OVATE* gene regulates the expression of many genes. In the transformation of *MuMADS1* and *MaOFP1* into the *ovate* mutant, the number of DEGs gradually decreased in the assemblies of the WT vs. *MuMADS1* transformants or WT vs. *MaOFP1* transformants. When *MuMADS1* and *MaOFP1* were cotransformed into the *ovate* mutant, the number of DEGs dropped to their lowest value. Secondly, the gene annotation and function classification (Figure 2), ‘carbohydrate transport and metabolism’ (64, 7.86%) in the WT vs. *ovate* mutant ranked highest. In the transformation of *MuMADS1* and *MaOFP1* to *ovate* mutant, ‘carbohydrate transport and metabolism’ was decreased to 44 (8.73%) and 10 (4.74%) in the assembly of WT vs. *MuMADS1* transformants and WT vs. *MuMADS1 + MaOFP1* transformants, respectively. We have summarized the distribution of DEGs regulated by MuMADS1, MaOFP1, MuMADS1 + MaOFP1 and OVATE based on the RNA‐Seq data as shown in Figure 6 and Table S7. OVATE regulated the largest number of target genes in the control of fruit quality, followed by the co‐expression of MuMADS1 and MaOFP1, which could nearly compensate for the *ovate* mutation. Either MuMADS1 or MaOFP1 could partially regulate the expression of genes involved in carbohydrate and cell wall metabolism to control fruit sugar accumulation and softening. *MuMADS1* is not a homolog of tomato *OVATE*. However, MuMADS1 shared almost all target genes such as *lysosomal beta‐glucosidase‐like* and *pectate lyase 8* with OVATE, MaOFP1 and MuMADS1 + MaOFP1. Similarly, MaOFP1 shared most target genes with OVATE and MuMADS1 + MaOFP1. MADS‐box genes have been shown to play a role in the formation of fruits (Tadiello *et al*., 2009). The suppression of the homologous *SEPALLATA1/2‐like* genes *MADS8* and *MADS9* in the fleshy fruit apple (*Malus* x *domestica*) leads to a change in ovary locule shape from a ‘tear drop’ to a more open triangular shape by strongly reducing the cortex layer (Ireland *et al*., 2013). Banana *MuMADS1* is an *AGAMOUS* MADS‐box gene. Our previous study indicated that *MuMADS1* is closely related to fruit ripening (Liu *et al*., 2009, 2015b), which suggests that *MuMADS1* plays an important role in controlling fruit quality. Therefore, an explanation for the partial compensation of the *ovate* mutation in tomato by MuMADS1 is that MuMADS1 itself plays a role in regulating fruit shape and quality in a similar manner as OVATE, by regulating sugar and cell wall metabolism, or by partially independent mechanisms. Our previous results demonstrated that MuMADS1 could interact with MaOFP1 to regulate banana fruit ripening (Liu *et al*., 2015b). The DEGs regulated by MuMADS1 + MaOFP1 were far greater in type and number than those of either MuMADS1 or MaOFP1, as shown in Figure 6 and Table S7, which might be the reason that overexpression of both genes in the *ovate* mutant results in an additive effect.

Fruit softening is a key trait for tomato fruit, and cell wall remodelling plays a major role in the textural changes and involves the coordinated expression of a large number of genes. In tomato, >50 cell wall structure‐related genes are expressed during fruit development (Tomato Genome Consortium, 2012; Minoia *et al*., 2016). Sucrose, a disaccharide, is an important end product of photosynthesis and is the primary carbon source for metabolism in the sink tissues of many plants. Sucrose must be cleaved either into UDP–glucose and fructose by sucrose synthase (SUS) or into glucose and fructose by invertase before it can be further metabolized (Dennis and Blakeley, 2000). The free hexoses, fructose and glucose, must then be phosphorylated by fructokinase (FRK) or hexokinase (HXK) before they can enter metabolic pathways (Stein *et al*., 2017). The tomato *SlFRK2* is essential for proper xylem development, and the xylem vessels in the stems of *SlFRK2* antisense plants have thinner xylem secondary cell walls with cells that are narrower and deformed (Damari‐Weissler *et al*., 2009). In this study, *plastidic hexokinase* (*Solyc04g081400.2*) was up‐regulated in the WT vs. *ovate* mutant, indicating that it contributes greatly to fruit cell wall biosynthesis in the *ovate* mutant, which was highly consistent with the firmer fruit observed in *ovate* than in WT. This up‐regulation could be suppressed by the co‐expression of *MuMADS1* and *MaOFP1* in *ovate*, which suggests that this gene may be regulated by *MuMADS1* and *MaOFP1*. The increased firmness associated with the *ovate* mutation might be due to its increased cell wall polysaccharide content, which may result in the reduction of soluble carbohydrates (composed of mainly sucrose, glucose and fructose; Figure 7).

β‐Glucosidase (BGL; EC 3.2.1.21) is a typical cellulase that acts synergistically to hydrolyse cell wall cellulose to glucose (Ma *et al*., 2016). The down‐regulation of β*‐glucosidase* (*Solyc07g063880.2*) might be the main factor accounting for the increase in fruit firmness and decreased glucose in the *ovate* mutant. However, this down‐regulation could be compensated for by the co‐expression of *MuMADS1* and *MaOFP1* in the *ovate* mutant, suggesting that *MuMADS1* and *MaOFP1* act synergistically to regulate the expression of β*‐glucosidase* (*Solyc07g063880.2*). In this study, another β*‐glucosidase* named *lysosomal beta‐glucosidase‐like* (*Solyc04g015560.2*) displayed a different expression pattern (Figure 5), suggesting that different members of the β‐glucosidase family with different properties exist in tomato fruit.

UDP–glucose 6‐dehydrogenase is an important enzyme involved in diverting UDP–Glc to cell wall synthesis (mainly hemicellulose) (Xue *et al*., 2008). Within the operon, a UDP–glucose 6‐dehydrogenase converts UDP–glucose to UDP–glucuronic acid and NADH in the presence of NAD+UDP–glucuronate 4‐epimerase 1‐like belongs to the family of short‐chain dehydrogenases/reductases (Broach *et al*., 2012). It converts UDP–glucuronic acid to UDP–galacturonic acid, the production of which is directly provided to Golgi‐localized galacturonosyltransferases during cell wall synthesis (Mølhøj *et al*., 2004). Therefore, the up‐regulation of *UDP–glucose 6‐dehydrogenase* (*Solyc02g067080.2*) and *UDP–glucuronate 4‐epimerase 1‐like* (*Solyc12g010540.1*) in the WT vs. *ovate* mutant may result in increased cell wall polysaccharide contents, which was consistent with the increased firmness observed in the *ovate* mutant. In the *MuMADS1* and *MaOFP1*‐transformed *ovate* mutant, the expressions of *UDP–glucose 6‐dehydrogenase* (*Solyc02g067080.2*) and *UDP–glucuronate 4‐epimerase 1‐like* (*Solyc12g010540.1*) decreased, which was consistent with the decreased firmness in the *MuMADS1 + MaOFP1* transformants (Figure 7).

Galacturonosyltransferase is a α‐1, 4‐galacturonosyltransferase that synthesizes homogalacturonan, the most abundant pectic polysaccharide (Atmodjo *et al*., 2011). The up‐regulation of three *galacturonosyltransferases* (*Solyc06g083310.2,* Solyc02g089440.2 and Solyc07g055930.2) in WT vs. *ovate* suggested that these genes contribute to the increased fruit firmness in the *ovate* mutant. This up‐regulatory phenomenon of three *galacturonosyltransferases* gradually disappeared as *MuMADS1* and *MaOFP1* transformed into the *ovate* mutation, which suggests that the expression of these genes is regulated by *MuMADS1* and *MaOFP1* (Figure 7).

**Figure 8:**
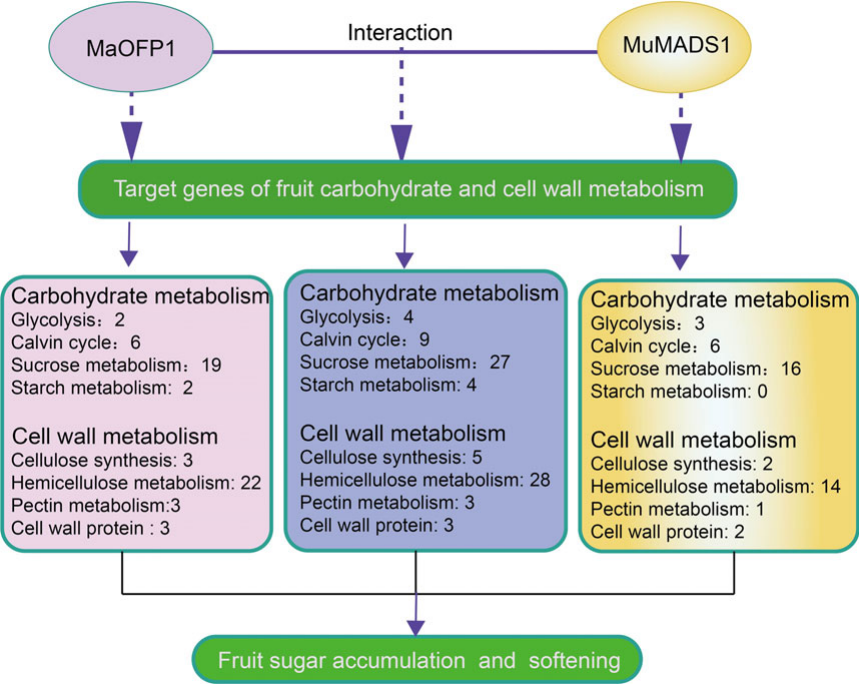
Model for the role of MuMADS1 and MaOFP1 in regulating fruit sugar accumulation and softening. The numbers of target genes involved in carbohydrate and cell wall metabolism regulated by MaOFP1 (the left box), MuMADS1 (the right box) and MuMADS1 + MaOFP1 (the middle box) have been summarized. Solid blue line, validated protein–protein interaction; solid blue arrows, validated regulation; dashed blue arrows, putative regulation.

Taken together, we suggest a mechanism by which MuMADS1 and MaOFP1 regulate fruit firmness and sugar accumulation. As shown in Figure 8, the putative target genes regulated by MuMADS1 could be placed into carbohydrate metabolism, including glycolysis (3), Calvin cycle (6), sucrose metabolism (16) and cell wall metabolism, including cellulose synthesis (2), hemicellulose metabolism (14), pectin metabolism (1) and cell wall protein (2). Starch metabolism (2) was added to the putative targets regulated by MaOFP1, and a number of other classes displayed a few more genes than those of MuMADS1. When MuMADS1 and MaOFP1 were co‐expressed, the number of genes involved in glycolysis, Calvin cycle, sucrose metabolism, starch metabolism and hemicellulose metabolism increased to 4, 9, 27, 4, 28, respectively, which was much higher than that of either MuMADS1 or MaOFP1. This is the first time that a mechanism for MuMADS1 and MaOFP1 in controlling fruit sugar accumulation and softening has been proposed.

## Experimental procedures

### Plant materials and treatments

Tomato *ovate* mutants (*S. lycopersicum,* LA3543) and wild‐type (*S. lycopersicum* ‘Ailsa Craig’, LA2838A) were kindly provided by the Tomato Genetics Resource Center (http://tgrc.ucdavis.edu). Plants were raised in a field environment in groups at the Institute of Tropical Bioscience and Biotechnology (Haikou, Hainan Province). Transgenic plants were produced to the T3 line, and just the homozygous transgenic plants were utilized in the quantitative analyses (Liu *et al*., 2015a). The fruits at various maturation periods were evaluated by labelling pollinated flowers: IMG [17 days after pollination (DAP)], MG, BR and RM (Bastías *et al*., 2011). Typical and transgenic plants were gathered, quickly frozen in liquid nitrogen and retained at −80 °C for later use.

### Constructs and genetic transformation

pCAMBIA1302 was digested by NcoI and SpeI, and then *MuMADS1* and *MaOFP1* were cloned into the multiple cloning sites to obtain the transformation vectors pCAMBIA1302‐*MuMADS1* and pCAMBIA1302‐*MaOFP1*. The two transformation vectors were introduced into *A. tumefaciens* EHA105. The two strains were used to infect the tomato *ovate* mutant together at a ratio of 1 : 1. The callus culture and genetic transformation procedures were based on the method described by McCormick *et al*. (1986).

### RNA extraction and cDNA synthesis

Total RNA from every one of the tissues utilized in this evaluation was removed with an altered cetyltrimethylammonium bromide (CTAB) technique (Wan and Wilkins, 1994).

First‐strand cDNA was combined with a SMARTTM PCR cDNA Synthesis Kit for reverse transcriptase (Clontech, Palo Alto, CA) based on the company's directions. The primers utilized for real‐time RT‐PCR are revealed in Table S8.

### qRT‐PCR analysis

Transcriptional changes in *MuMADS1* and *MaOFP1*, as well as key genes responding to fruit quality formation in transgenic lines and controls, were determined via qRT‐PCR analysis on a Stratagene Mx3000P Real‐Time PCR system with SYBR^®^ Premix Ex Taq™ (TaKaRa, Japan). The PCR amplification set up for every one of the reactions was: 10 min at 95 °C, then 40 cycles of 10 s at 95 °C, 15 s at 50 °C and 30 s at 72 °C. The typical expressions of the target genes were quantified by the 2^−ΔΔ^CT method (Livark and Schmittgen, 2001). *18S* rDNA (accession number: X51576.1) was used as the internal control to normalize the expression of the target genes in tomato.

### Southern blot analysis

The Southern blot analysis was conducted based on Liu *et al*.'s technique (2015a).

### Measurements of fruit quality‐related physiological indexes

Upon harvesting, three fruits per treatment were sampled for measurements among the marked fruits. After weighing, the fruit sample was measured for longitudinal and transverse diameter (cm) using a Vernier calliper (Li *et al*., 2016). The fruit shape index was analysed by longitudinal/transverse diameter.

The taste and nutritional properties of the first ripe fruit harvested from the first truss of each tagged plant were determined. The fruit was sliced and blended after removing the skin and seeds. A hand‐held ATAGO‐P32 temperature compensated refractometer (ATAGO Co. Ltd, Tokyo, Japan) was used to directly read the % soluble solids (as Brix) of the blended fruit at room temperature (Pieper and Barrett, 2009).

Fruit solidity was quantified based on Li *et al*.'s technique (2013).

The sugar (fructose, glucose and sucrose) contents were analysed using high‐performance liquid chromatography (HPLC; Waters, Milford, CT).

### De novo transcriptome assembly and annotation

RM fruits of different transgenic lines including M (*MuMADS1* transformants), O (*MaOFP1* transformants) and MO (*MuMADS1 *+* MaOFP1* transformants), as well as WT and *ovate* were collected to extract total RNA using the plant RNeasy extraction kit (TIANGEN, Beijing, China) for transcriptome analysis. The sequencing was performed with an Illumina GAII following the manufacturer's instructions, with three replicates for each sample. The average sequencing depth was 5.34X. Using the FASTX‐toolkit, adapter sequences in the raw sequence reads were removed. Clean reads were generated after examining the sequence quality and removing low‐quality sequences using FastQC. Using Tophat v. 2.0.10, clean reads were mapped to the tomato genome (*S. lycopersicum* L, 2n = 24). The transcriptome assemblies were performed by Cufflinks (Trapnell *et al*., 2012). Gene expression levels were calculated as Reads per Kilobase of exon model per Million mapped reads (FPKM). DEGseq was used to identify DEGs (Wang *et al*., 2010). Significant DEGs were screened using DESeq software (Anders and Huber, 2010). The corrected *P*‐values from this method accounting for multiple tests used the key factor, which was false discovery rate (FDR). FDR <0.01 and |log2 (fold change)| > 1 or <−1 were set as the thresholds for differential gene expression. Fold changes in the expression levels between samples were used as the criteria in the screening process. The unigene sequences were searched against the following public databases: NR database (Deng *et al*., 2006), Swiss‐Prot (Apweiler *et al*., 2004) GO database (Ashburner *et al*., 2000), COG database (Tatusov *et al*., 2000) and KEGG (Kanehisa *et al*., 2004).

## Conflict of interest

The authors declare that they have no conflicts of interest.

## Supporting information


**Figure S1** Identification of transgenic tomatoes.

**Figure S2** GO classification. The DEGs corresponded to three main categories: “biological process”, “cellular component” and “molecular function”. The left‐hand *y*‐axes indicate the percentage of genes.

**Table S1** RNA‐Seq analysis.
**Table S2** Mapped results of RNA‐Seq and reference genome.
**Table S3** Annotated number of DEGs.
**Table S4** Analysis of GO classification.
**Table S5** Analysis of KEGG classification.
**Table S6** The expression data of the DEGs in different assemblies.
**Table S7** The DEGs regulated by OVATE, MuMADS1, MaOFP1 and MuMADS1 + MaOFP1.
**Table S8** The primer sequences used for qRT‐PCR.
