# Supplementary figures and images for: MuMADS1 and MaOFP1 regulate fruit quality in a tomato ovate mutant
Source: Plant Biotechnol J. 2017 Nov 2;16(5):989–1001. doi: 10.1111/pbi.12843 (PMC5902769; doi:10.1111/pbi.12843)

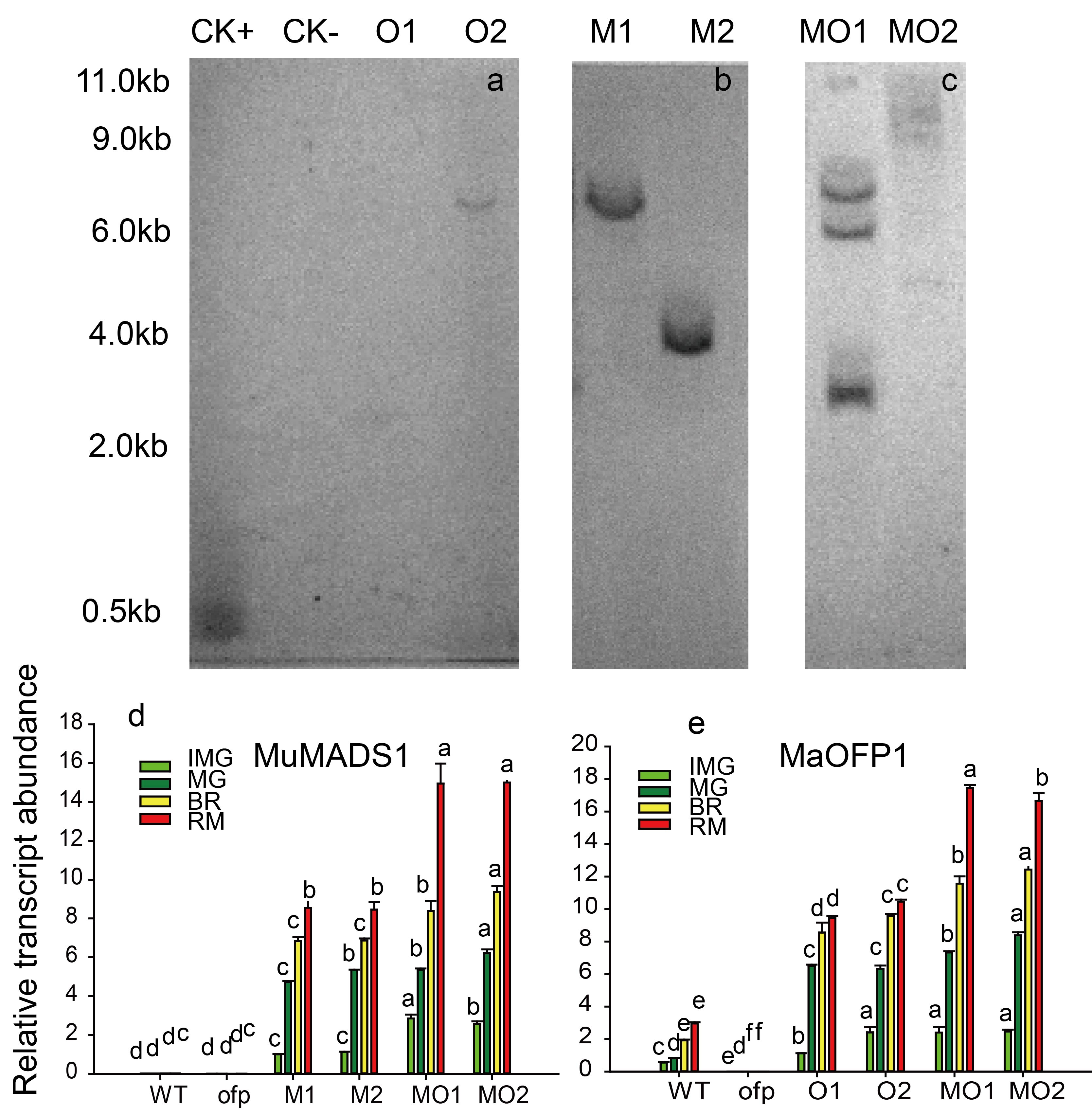

Supplement: Supplementary file 1 — Figure S1 Identification of transgenic tomatoes. [file PBI-16-989-s003.jpg]

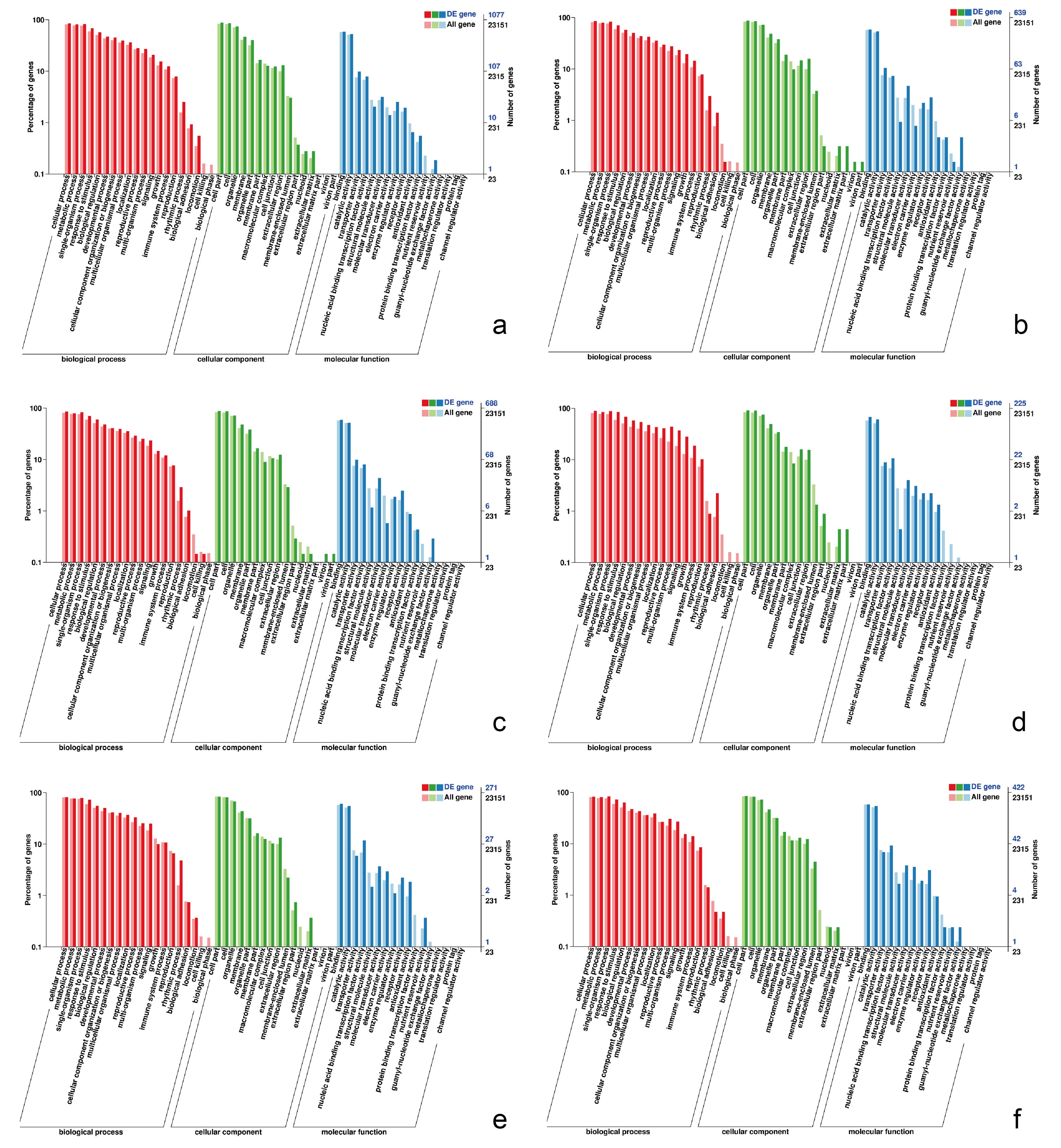

Supplement: Supplementary file 2 — Figure S2 GO classification. The DEGs corresponded to three main categories: “biological process”, “cellular component” and “molecular function”. The left‐hand y‐axes indicate the percentage of genes. [file PBI-16-989-s002.jpg]
